# Building medical physics continuing education programming tailored to the site-specific needs in low- and middle-income countries: an initial framework

**DOI:** 10.3389/fmed.2025.1634679

**Published:** 2025-10-29

**Authors:** William Swanson, Nataliya Kovalchuk, Iyobosa Uwadiae, Matt Goss, Serhii Brovchuk, Ruslan Zelinskyi, Victoria Ainsworth, Stephanie Parker, Stephen Avery, Wil Ngwa, Kelly Kisling

**Affiliations:** ^1^Emory University, Atlanta, GA, United States; ^2^Stanford University, Stanford, CA, United States; ^3^University College Hospital, Ibadan, Nigeria; ^4^Allegheny Health Network, Pittsburgh, PA, United States; ^5^LISOD Israeli Oncology Hospital, Pliuty, Ukraine; ^6^O.O. Shalimov National Scientific Center of Surgery and Transplantation, Kyiv, Ukraine; ^7^National Cancer Institute, Kyiv, Ukraine; ^8^University of Massachusetts Lowell, Lowell, MA, United States; ^9^Atrium Health Wake Forest Baptist High Point Medical Center, High Point, NC, United States; ^10^University of Pennsylvania, Philadelphia, PA, United States; ^11^Johns Hopkins University, Baltimore, MD, United States; ^12^University of California, San Diego, San Diego, CA, United States

**Keywords:** medical physics education, LMICs (low and middle income countries), clinical training, cancer, radiation oncology

## Abstract

Low- and middle-income countries (LMICs) are significantly underserved in cancer management resources, contributing the majority of annual cancer incidence and mortality globally. Many patients in LMICs have limited accessibility to radiotherapy, which serves as a standard cancer treatment for many patients, due to lacking infrastructure to support the patient population and trained professionals to deliver treatment. Recent efforts have been made to supplement foundational medical physics knowledge in LMICs by providing continuing education opportunities to establish strong educational leaders at the local level. While high-income countries have ample resources to support well-established education and training standards, it is important to adapt the curricula in a resource-responsible manner. This education and training should be relevant and appropriate to the clinical setting in LMICs. This article collates the experience of members of the American Association of Physicists in Medicine and international collaborators to develop and administer optimized medical physics education with limited resources. The framework presented here is intended to be used as a preliminary guide to developing education and training programs specific to the needs of medical physicists in LMICs.

## Introduction

Substantial cancer management disparities in low- and middle-income countries (LMICs) demand investment of more resources, including the creation of additional healthcare facilities and installation of new equipment, the adoption of new cost-effective therapeutic techniques, and the education and training of local professionals. According to the World Health Organization Global Cancer Observatory, there were more than 10.2 million new cancer cases and more than 6.8 million cancer-related deaths across LMICs in 2022 alone (1). This is projected to increase to over 17.1 million and 13.4 million incidences and deaths, respectively, by 2050 (1). The cancer burden among LMICs constitutes half of all incidences and 70% of all cancer-related deaths globally (1). Radiotherapy serves as a major modality for treatment of cancer, however access is scarce in LMICs. According to the International Atomic Energy Agency Directory of Radiotherapy Centers, there is 1 radiotherapy unit for every 1.17 million people in LMICs, whereas in high-income countries (HICs) there is 1 unit for every 120 thousand people (2). With cancer incidence drastically increasing and mortality nearly doubling by 2050 across LMICs, access to cancer management resources is crucial for these underserved populations. The 2022 Lancet Oncology Commission laid out a recommended course of action to address these disparities in Sub-Saharan Africa, highlighting healthcare workforce education and training as a crucial element to improving healthcare equity and access (3).

The Global Needs Assessment Committee (GNAC), from the American Association of Physicists in Medicine International Council, recently reported on a global survey of LMIC cancer centers that assessed local needs for cancer management. The report demonstrated that among the 175 respondents across 48 LMICs, the second greatest need was an increase in qualified staff, the first being new and updated equipment (4). 58% of respondents indicated a need for clinical training and 45% indicated a need for a formal course specifically in medical physics (4). Additionally, 49% of respondents reported that training provided by equipment suppliers was insufficient for clinical practice (4).

Common efforts to increase capacity in cancer care include the adoption of new cost-effective treatment techniques, the installation of additional treatment units and equipment, or the construction of entirely new healthcare facilities. These endeavors have significant training requirements for personnel, which can pose a significant challenge in some LMICs where access to formal education or training programs is lacking. Medical physics training in LMICs may range from structured degree or residency programs as is common in HICs to less formalized on-the-job apprenticeships, and some countries may not even recognize medical physics as a profession (5, 6). A structured and sustainable educational system would enable a growing expert workforce to grow and keep up with the expansion and improvement of physical infrastructure.

While developing additional initial education and training programs would be ideal to bring new professionals into the field, continuing education programs (CEPs) can be used to supplement pre-existing formal education programs to improve the competency and confidence of working professionals. It is important for medical professionals to maintain knowledge in a growing field, improving the quality of care and maintaining certification. Professionals seek continuing education through refresher courses, webinars on new practices and innovations, and medical conferences. CEPs serve well as pathways to enable professionals as high quality instructors and mentors for others in the field. In addition to improving knowledge and teaching new skills, continuing education has been associated with increased employee retention and career satisfaction (7). Implementing and sustaining CEPs in LMICs can be challenging for many reasons. When programs are collaborations between HICs and LMICs, barriers to success have been found to include Western bias and cultural differences while enablers include fostering relationships, meeting local needs, and developing participants to become future CEP educators (8). While the role of CEPs in LMICs for other healthcare fields has been studied (9, 10), to our knowledge no published recommendations related to the role of CEPs for medical physicists in LMICs exists. The International Atomic Energy Agency (IAEA) and International Organization of Medical Physicists (IOMP) have training resources and webinars available to professionals both in HICs and LMICs. The CEPs described in this work provide additional options for accessibility and are curated specifically to the needs in the target region to optimize time and resources.

The purpose of this work is to act as a guide to develop a CEP in LMICs to supplement pre-existing education with a curriculum specifically tailored to the needs of the local professionals. The intent of these programs is to expand access to high quality training which meets international standards such as those provided by IAEA and the IOMP. This guide will walk through the framework development process including the presentation of two CEP case examples coordinated by the American Association of Physicists in Medicine (AAPM), followed by discussions of sustainable education practices, identifying of the target audience’s goals and developing a curriculum, logistical challenges and limitations on CEP delivery, acquiring certification or endorsement for the CEP, and optimally allocating resources. The guidelines developed in this manuscript were based on the collective experience of the authors.

### AAPM collaborations: NAMP and HUG/UAMP case examples

In an effort to address the need for education, two recent medical physics continuing education courses, endorsed by the American Association of Physicists in Medicine (AAPM) and accredited by the Commission on Accreditation of Medical Physics Education Programs (CAMPEP), were successfully administered to groups within low- and middle-income countries (LMICs). The courses were a collaboration between the Global Health Catalyst Global Oncology University (GOU), the AAPM, the Nigerian Association of Medical Physicists (NAMP), the Ukrainian Association of Medical Physicists (UAMP), and the Help Ukraine Group (HUG). The pilot course for NAMP, funded partially by AAPM and GOU, successfully awarded CAMPEP continuing education credits to 53 medical physicists working professionally in Nigeria (with 76 enrolled in the course) (11). To improve participant performance in meeting CAMPEP criteria, future iterations of the course will explore the implementation of asynchronous learning resources to aid in schedule conflicts with course attendance and more engaging learning methods during live lectures. The NAMP course focused on harmonizing medical physics education across Nigerian institutions and preparing the attendees for clinical certification through the International Medical Physics Certification Board (IMPCB). The NAMP course development was supported in collaboration with the Secretary-General of NAMP, Dr. Iyobosa Uwadiae. In the second course for HUG/UAMP, funded partially by HUG and GOU, 131 attendees (100 practicing physicists and 31 students, 44% of which had prior formal medical physics education) were enrolled in virtual parts 1 and 2 of the course and 61 attendees were enrolled in the practical part 3 of the course focused on the topics of linear accelerator acceptance and commissioning, treatment planning, and quality assurance program development (12, 13). The UAMP course development was supported in collaboration with the President of UAMP, Ruslan Zelinsky.

The NAMP and HUG/UAMP (parts 1 and 2) courses were delivered as an online-only course. Weekly and twice weekly lectures, respectively, were delivered over Zoom and the course was organized on a virtual learning management system (TalentLMS). Each session incorporated active learning through virtual polling systems and open discussions between participants and instructors. Sessions were divided among several volunteer instructors recruited through the network of AAPM members with expertise in each lecture topic and experience in education. Pre-lecture reading material assignments and post-lecture multiple choice knowledge assessments were incorporated into the course design. Pre- and post- CEP competency assessments were administered to measure the effectiveness and educational impact on the participants. As part of CAMPEP-accreditation, participants were required to complete knowledge assessments with 80% success to earn credit for each session. The instruction and administration of these courses were solely by volunteers through the collaborating AAPM committees: The NAMP course included 1 course director, 3 course administrators, and 16 instructors, and the HUG/UAMP course parts 1 and 2 included 1 course director, 2 course administrators, and 34 instructors. The HUG/UAMP course also used the AI-driven synchronous translation Zoom feature to effectively transmit the knowledge in English to the course participants with English language barriers (61% requiring translated subtitles). The HUG/UAMP course also engaged the volunteers from the Ukrainian CEP participants to edit the AI-driven subtitling in the recorded lectures (evaluated to be acceptable quality translation by 72.6% of the participants). While the teams developing these courses have built a framework of resources to provide these services to LMICs, it is important to build sustainable efforts to ensure continued education and training in the future, including training led by local experts.

### Considerations for sustainability

When establishing a CEP for LMICs as an external entity, sustainability of the program is an important consideration. Collaboration with local experts who have on-the-ground experience and intimate knowledge of the clinical context of the region is recommended. By taking local context into account, the CEP’s framework can be constructed such that the operation and execution of the CEP can be transitioned to local professionals. The framework of the CEP should be designed to sufficiently educate and train the participants to perform the necessary tasks with the competence and confidence of an instructor. The external entity (e.g., institution, professional organization, etc.) can then shift to a role of administration and management and eventually transition the CEP to LMIC local professionals to run independently. As the responsibility for instruction is transferred to local professionals, education and training can be further tailored to the evolving needs of the regional clinics. Additionally, as the field progresses, periodic future intervention may be considered to update CEP content.

### Continuing education objectives and motivations

The administration of the CEP is for the benefit of the participants. To deliver those benefits, the CEP should be developed with the goals of its participants in mind. While the initiating external entity may already have set standards for a CEP, it would be unwise to directly apply the teaching and training without taking the local context into account. Therefore, the goal(s) of the local participants should be assessed. Example goals include a review of fundamental practices in preparation for board certification, education on specific topics which may be poorly understood or infrequently referenced in clinical work, or the introduction of new topics due to new equipment or protocols being introduced in local clinics.

To identify the current academic level and motivations of CEP participants, a needs assessment is required. This assessment can take many forms but will be discussed here in the form of an online survey administered by the CEP administrative team. A sample template is provided in Supplementary material. The survey should collect demographic information about the background of the participants including years of pre-work training and time spent in the profession. This information will give a sense of the participating group’s experience and career stage and allow the program developer to determine baseline expectations. To prevent assumptions about the participants’ baseline knowledge and experience, additional information should be gathered to provide greater context. One method to accomplish this would be through including a high-level curriculum in the survey which assesses how knowledgeable and confident the participants are in those subject areas. Accrediting curricula, such as those provided by the IAEA and IOMP used to accredit education and training programs for medical physicists, provide an education standard from which the CEP can be tailored to local needs. This provides context on the current practices and available technology at those sites. It also creates a priority list of those topics for which the allotted program time can be optimized. Designing key metrics into the survey and deploying the survey both before and after the CEP would provide an opportunity to assess impact of the CEP. As self-assessments are inherently flawed due to the participant not necessarily knowing the full scope of what is being assessed, a pre-CEP competency examination could also be used to evaluate knowledge gaps and where competence and reported confidence do not agree. It is important to balance the time refining the course with avoiding delays in initiating.

Another method of prioritizing education and training material is through creating an open dialogue with key stakeholders such as institutional leaders and representatives of potential CEP participants. Ideally, those representatives also are actively practicing professionals such that they have direct connection to the field. Important elements of discussion include the audience demographic (career stage, experience level), current clinical practice (equipment available, disease sites treated), and potential major changes to the clinic (new protocols, new equipment installations). Such dialogue can provide valuable information on the motivations of the cohort as well as the timelines required for adequate training. For example, the recent HUG/UAMP course was focused on training for accelerator acceptance, commissioning, and treatment planning as that cohort was anticipating a transition from Co-60 teletherapy units to megavoltage accelerators over the next few years (12, 13). An open dialogue with representatives of the cohort may result in a clearer picture of the clinical needs than what a survey alone can achieve. Additionally note that the needs not only define the scope of the content, but also the size of the audience. With the intent of sustainability and using a “train-the-trainer” model, aiming for a multi-institutional to regional CEP would be recommended.

### Program delivery and evaluation

The scope of education and training of a CEP should be specific to the setting and needs of the participants. The CEP may include a combination of learning methods including independent study, lectures, and practical exercises. The curriculum aspects may be provided in various formats such as a resource library which the participant may use to access course material, guided study through an assigned mentor, online learning platforms to interact with learning material and instructors, and on-site hands-on training in a safe environment to simulate clinical practices. The size of the CEP cohort should be considered to determine the most effective and efficient curriculum delivery strategies. If a “flipped classroom” style of learning is involved, a large cohort may warrant dividing into smaller groups which would require additional administrative effort to be effective (14). Engagement of participants through active learning methods may require additional administration and technical resources. Alternatively, for a relatively small cohort, professional mentorship could be the primary mode of training. A personally assigned mentor can guide the student through didactic readings, practical exercises, and/or a special topics project. The cohort size and learning methods should be selected based on the resources available.

CEP participant enrollment forces several questions: “What is the capacity for CEP enrollment?,” “How are participants recruited?,” “What criteria must the participant satisfy to enroll in the CEP?” Enrollment capacity is determined by the physical and technical constraints of the CEP platform (physical classroom size or digital platform user limits), the practicality of education delivery methods (higher capacity for large lecture groups and lower capacity for active learning), and budget constraints (certifying credit fees, platform user subscriptions, etc.). Recruitment of participants can be achieved by circulating advertising material through key stakeholders of the target audience of the CEP (institutional, regional, or national leaders within target LMICs). If recruitment is anticipated to exceed enrollment limits, an application process should be implemented, and the appropriate selection criterion must be established prior to opening application registration (education degree level, years of experience, professional organization membership, etc.).

Lastly, the effectiveness of the CEP should be measured after completion in order to assess the participants’ competency and to identify opportunities for improvement. A competency examination at the end of the CEP commonly serves as a method to evaluate participants, however depending on the goals of the program, the evaluation could take the form of a live practicum or participant presentation. The effectiveness of the CEP could be measured through various methods: baseline competency of the participants based on the criteria of the program administrators and/or accrediting bodies, competency improvement of the participants by comparing pre- and post- CEP assessments, and participant surveys to provide feedback on how the CEP influenced their own confidence, performance, and professional development. These performance metrics demonstrate the impact of the CEP among its participants and can be used to justify further expansion and improvement of the program. Additionally, the post-CEP survey may be used to recruit participants to act as administrators and instructors for future iterations.

### Certification and endorsement

A course completion certificate is a typical expectation after participation. The certificate not only serves as a record of participation, employers may also use the certificate to support career advancement. To provide additional weight and value to the certificate, accreditation (with approved continuing education credits) or endorsement can be acquired through professional organizations which either provide or assess education and training efforts, such as the CAMPEP and the AAPM, respectively, in the United States. Accreditation and endorsement may be beneficial in countries seeking professional recognition. The process of acquiring accreditation or endorsement will differ between endowing organizations but will likely include a review of the program to ensure it meets the appropriate standards. The criteria for accreditation or endorsement should be sought early in the course development process so that it can be considered in the course design. Additionally, regionally appropriate accreditation and endorsement which holds value for the target audience should be sought. CEP administrators should ensure that the criteria for earning continuing education credits are satisfied by participants. Accrediting organizations will typically require a fee with each certificate provided so the cost should be included in the CEP budget.

### Resource allocation

Time, funding, and logistics are important to manage when administering a CEP. Record keeping for participants, and especially for large cohorts, can be done using a digital learning-management-system (LMS). LMS platforms typically have features which allow the administrator to enroll participants, distribute learning material and assignments, and track progress and evaluations. The features provided by an LMS can substantially reduce the workload of CEP administrators. A regularly maintained LMS ensures a smooth and reliable experience for both the participants and instructors, minimizing time spent navigating the LMS. The cost of maintaining an LMS can run up to several hundred dollars monthly. Executing a CEP requires administrative efforts to build and initiate the CEP as well as continuously support and maintain it.

The time committed by administrators and instructors must be considered when establishing the logistics of the CEP, such as session frequency, cohort size, if continuing education credits will be administered, and program complexity. Instructors will require time to prepare lesson material such as lecture slides, pre-lecture reading material, and post-lecture assignments and evaluations. Instructors with prior experience may be able to use their own prior teaching material but may also need to modify it to add focus to the equipment available in LMIC facilities. Expertise and content should also be vetted for quality and relevance. For example, CAMPEP requires instructors to submit a statement of interest highlighting their experience and expertise as an educator for a specific topic. Administrators will require time to coordinate with instructors and students regarding technical support using the LMS and technical presentation systems such as audio-visual systems in a lecture hall or streaming platforms in a virtual classroom. The roles of the CEP administrator may include but are not limited to management of learning management systems, technical support for instructors, learning material distribution, evaluations, and instructor and participant coordination. A method to reduce the time commitment on instructors would be to have the course be taught by a series of guest lecturers. Each lecturer would have the commitment of only a few didactic sessions as opposed to an entire course. However, additional efforts from the administrative team will be required to “on-board” each instructor to the course. Additional support from the administrative team will reduce the efforts of instructors, allowing them to focus more on course instruction. However, it is worth nothing that coordinating between several lecturers may introduce challenges with material continuity and overlap. Administrators and instructors should be compensated for the time committed to their responsibilities for the CEP. Volunteers can be compensated with professional service acts which can contribute toward professional advancement, financial incentives (hiring), or other methods, as appropriate. As sustainability is a key component to a CEP, participants local to the cohort’s region should be encouraged to volunteer as administrative and teaching assistants in future iterations of the CEP, preparing them to eventually assume full responsibilities of growing and maintaining the CEP. This opportunity would offer a pathway to become a regional expert in medical physics education.

## Conclusion

The recommendations provided in this article may serve as a guide to building a sustainable CEP framework for medical physicists in LMICs. The presented pilot CEPs by the AAPM act as a proof of concept for building a new “LMIC-collaborative” CEP, for which new iterations with previous participants as administrators and instructors are currently underway. While the tailored content offers much value to the audience when made with the local context in mind, future iterations of those pilot CEPs will explore offering greater access to existing resources to LMICs, such as the AAPM virtual library, and offering pre-recorded lecture material from previous LMIC-tailored iterations. The recommendations, summarized in Table 1, address various aspects of developing a CEP from performing a needs assessment of the target audience, managing logistics and resources of delivering the CEP, and measuring impact within the setting of resource-limited regions. The authors hope that by prioritizing the immediate needs of the target audience, gaps in education and training can be more quickly and efficiently satisfied. The guide not only aims to provide “patchwork” training on new equipment or techniques clinics wish to implement in their practices but also creates a pathway to supplementing local education systems in LMICs to enhance local expertise. Local experts supported by a CEP framework can then sustainably provide high quality training, leading to increases in skilled workforce capacity. The authors encourage the support of efforts toward improving radiation oncology workforce capacity to increase radiotherapy access in LMICs where patient loads are significant.

**Table 1:** Overview of recommendations for building medical physics CEPs in LMICs.

Aspect of CEP	Recommendations
Considerations for sustainability	Acquire feedback from the participants and instructors regarding the Continuing Education Program (CEP). The feedback should be used to improve future iterations of the CEP and measure the impact on the participants. Further follow-up of the participants is recommended to determine long-term impact of the CEP. Some key indicators include the participant’s occupational performance, professional advancement, board certification, status, etc. Identify participants which would be suitable and interested in acting as a CEP administrator or instructor for future iterations, sustaining the CEP among local professionals.
Continuing education objectives and motivations	Open a dialog with the stakeholders of the target audience to determine the objectives to achieve by execution of the CEP. Develop a baseline curriculum based on the appropriate pre-existing standards. The International Atomic Energy Agency (IAEA) and International Organization of Medical Physicists (IOMP) guides serve as excellent resources for medical physics standards. Perform a needs assessment of the target audience and determine potential gaps in education and training among the cohort. Determine and apply for regionally valuable accreditation or endorsement. Optimize the curriculum to the needs of the target audience given the identified educational gaps, discussed objectives, and accreditation/endorsement requirements.
Program delivery and evaluation	Determine the learning methods which best suit the needs of the curriculum while keeping in mind the limitations of the CEP (funding, personnel, logistics, etc.). Utilize a learning management system to manage the CEP (track participant progress, disseminate learning material, communicate and coordinate with participants and instructors, etc.). Evaluate the participants’ progress in competence and confidence throughout the course. The post-CEP evaluation should be compared to the initial needs assessment to measure effectiveness of the CEP.
Certification and endorsement	Seek accreditation by entities relevant and valued by the targeted audience. Adhere to all requirements necessary for accreditation credits and/or endorsements by external entities. This may include fees, subject material to be covered, and evaluation criteria.
Resource allocation	Recruit administrators and instructors for the CEP. Compensation can vary based on responsibilities, from acts of professional development to monetary incentives. Keep in mind the upkeep responsibilities depending on the size of the participant cohort, budget, and available personnel. A multi-instructor course may increase administrative needs for CEP coordination.

## Data Availability

The original contributions presented in the study are included in the article/Supplementary material, further inquiries can be directed to the corresponding author.
