# Supplementary material for: Building medical physics continuing education programming tailored to the site-specific needs in low- and middle-income countries: an initial framework
Source: Front Med (Lausanne). 2025 Oct 29;12:1634679. doi: 10.3389/fmed.2025.1634679 (PMC12605069; doi:10.3389/fmed.2025.1634679)
Supplement: Supplementary file 1 [file Data_Sheet_1.docx]

Example Continuing Education Program Needs Assessment and Participant Intake Form

1. Please provide your contact information
   1. [Name]
   2. [Email]
2. In what time zone are you located?
3. Describe your career stage as a medical physicist.
   1. Student
   2. Resident/Trainee
   3. Working Professional (non-trainee)
4. How many years have you been working in the field as a professional (non-trainee)?
   1. [number of years]
5. With which institution are you affiliated?
   1. [Institution Name]
   2. [Institution City]
   3. [Institution Country]
6. How would you classify your institution?
   1. Public/Government Hospital
   2. Private/Community Hospital
   3. Medical School/University Hospital
   4. Academic Institution (non-hospital)
   5. Other [ _____ ]
7. Are you currently practicing medical physics in a clinical setting?
   1. Yes
   2. No
8. How many medical physicists work at your institution?
9. What is your practicing specialty? [check all that apply]
   1. Radiation Therapy
   2. Diagnostic Imaging
   3. Nuclear Medicine
   4. Other [ _____ ]
10. What is your highest degree level?
    1. PhD
    2. Masters
    3. Bachelors
    4. Other [ _____ ]
11. In what field of study is your degree?
    1. Medical Physics
    2. Other [ _____ ]
12. From where did you receive your degree and when did you graduate?
    1. [Institution Name & Graduation Year]
13. From where did you receive clinical training?
    1. [Institution Name]
    2. I did not receive formal clinical training outside of my job.
14. What continuing education activities have you participated in?
    1. “Conference A”
    2. ...
    3. “Webinar A”
    4. ...
    5. “Course A”
    6. ...
    7. “Other A”
    8. ...
15. What certifications do you hold in medical physics? [check all that apply]
    1. International Medical Physics Certification Board (IMPCB)
    2. “Certification Board B”
    3. “Certification Board C”
    4. ...
    5. No certifications
    6. Other [ _____ ]
16. What professional organization memberships do you hold in medical physics? [check all that apply]
    1. “Organization A”
    2. “Organization B”
    3. “Organization C”
    4. ...
    5. No Memberships
    6. Other [ _____ ]
17. This program will follow the following schedule: [describe schedule and time commitment of the program]. Please acknowledge your acceptance of this time commitment.
    1. Yes, I accept the full time commitment associated with this program.
    2. Yes, I am able to commit to participating in most of the program.
    3. Yes, but I only plan on participating in the relevant parts of the program.
    4. No, I do not have enough time to participate in the program
18. This program is currently only offered in the following languages: [insert each language the program will be available in]. Please acknowledge your acceptance of this format.
    1. Yes
    2. No
19. Do you require continuing education/professional development credits? If so, please indicate what type?
    1. International Organization of Medical Physicists (IOMP)
    2. “Organization B”
    3. “Organization C”
    4. ...
    5. No
    6. Other [ _____ ]
20. How confident do you feel in your understanding and ability to apply the following topics? [1 = Not confident at all, 2 = A little confident, 3 = Moderately confident, 4 = Very confident, 5 = Extremely confident]
    1. “Topic A” [1-5]
    2. “Topic B” [1-5]
    3. “Topic C” [1-5]
    4. ...
21. Please indicate additional related topics which you feel need to be addressed:

Example Post Continuing Education Program Evaluation Form

1. On a scale of 1 to 5 (1 being poor and 5 being excellent), how would you rate these aspects of each lecture?
   1. Learning Objective Appropriateness
   2. Information Usefulness
   3. Presentation Quality
   4. Lecture Note Quality
   5. Reading Material Quality
   6. Assignment/Examination Quality
   7. Instructor Competence
   8. Opportunities for Engagement
   9. Smoothness of Administration
   10. Worthiness of Repeating
2. On a scale of 1 to 5 (1 being no impact and 5 being major impact), how would you rate the positive impact of this continuing education program on the following?
   1. Your medical physics knowledge
   2. Your clinical practice
   3. Your career
   4. Your plans to pursue clinical certification
3. On a scale of 1 to 5 (1 being poor and 5 being excellent), how would you rate these technical aspects of the continuing education program?
   1. Learning Management System/Course Website
   2. Webinar Platform
   3. ...
4. Would you recommend this course to your colleagues?
   1. Yes
   2. No
5. How confident do you feel in your understanding and ability to apply the following topics? [1 = Not confident at all, 2 = A little confident, 3 = Moderately confident, 4 = Very confident, 5 = Extremely confident]
   1. “Topic A” [1-5]
   2. “Topic B” [1-5]
   3. “Topic C” [1-5]
   4. ...
6. Please provide any comments and feedback on the continuing education program, including positive aspects of the program, areas to improve, and material you would like covered in greater detail.
